# Evolution of Life Satisfaction Throughout the Gestation Process and at Different Postpartum Stages

**DOI:** 10.3390/bs15101390

**Published:** 2025-10-14

**Authors:** María Crespo, Miri Kestler-Peleg, Patricia Catalá, Celia Arribas, Cecilia Peñacoba

**Affiliations:** 1 Department of Psychology, Universidad Rey Juan Carlos, Avda. de Atenas s/n, 28922 Alcorcón, Madrid, Spain; maria.crespo.diaz@urjc.es (M.C.); patricia.catala@urjc.es (P.C.); c.arribasj.2020@alumnos.urjc.es (C.A.); 2 School of Social Work, Ariel University, Ariel 40700, Israel; mirikp@ariel.ac.il

**Keywords:** life satisfaction, perinatology, postpartum, motherhood, mental health

## Abstract

**Background**: Life satisfaction during the perinatal period has gained increasing attention as a relevant indicator of mental health, providing a more complete view of women’s adaptation to motherhood. **Methods**: This study examines the evolution of life satisfaction across four time points: the third trimester of pregnancy (T1), 8 weeks after birth (T2), 5 months after birth (T3) and 5 years postpartum (T4). A total of 231 women participated in this longitudinal study. Participants completed an ad hoc questionnaire for sociodemographic data and standardized self-report measures assessing different personality variables (attachment style, social support, maternal self-efficacy and positive/negative affect) at T1 as well as the Satisfaction with Life Scale (SWLS) (T1–T4). Statistical analyses were performed in order to evaluate the relationships between variables (Student’s test, ANOVA, Pearson’s correlation), and a linear regression analysis was conducted to explore the contribution of psychosocial variables at each time point. Additional subgroup analyses (employment status and parity) were carried out. **Results**: Results showed that at the first and second time points, life satisfaction was maintained, but five months after birth (T3), it decreased, and then it changed again five years after birth, increasing to its highest level. The psychosocial variables significantly associated with changes in life satisfaction varied across time points. Differential profiles are also observed depending on the subgroup. **Conclusions**: These findings suggest that monitoring these variables throughout the perinatal period may help identify women at risk and guide interventions in addition to preventive programs aimed at promoting well-being during the perinatal period and into motherhood.

## 1. Introduction

Life satisfaction is a subjective measure of well-being reflecting a global cognitive appraisal of one’s life (Diener et al., 1985). This appraisal is defined relative to personal standards and values rather than to any single external indicator (Diener et al., 2018). As a cognitive component of well-being, life satisfaction differs from affective dimensions and is generally stable, though influenced by significant life events. Various personal and contextual factors contribute to life satisfaction, including personality traits, health state, and sociodemographic conditions such as socioeconomic level, education, or marital status (Dziurka et al., 2021). Some studies highlight the role of psychosocial factors and the socioeconomic and health status (Mao et al., 2024; Quick et al., 2023), while others examine associations with financial stability and place of residence (Akkiraju & Rao, 2025; Prati, 2024). These findings underscore the multifactorial nature of life satisfaction, shaped by both individual characteristics and socio-familial contexts. Recent longitudinal evidence indicates that life satisfaction shows variability across the perinatal period, with patterns differing across populations. However, the number of studies remains limited, which highlights the need for further research examining these trajectories in diverse sociocultural contexts (Quick et al., 2023).

Studying life satisfaction during transitional life periods such as pregnancy and postpartum is particularly relevant. Moreover, pregnancy has been conceptualized as a significant life event, given its profound physical, psychological, and social implications (Van Haeken et al., 2020). These phases require adaptation efforts from women, so life satisfaction may fluctuate during these phases. These life changes and the ways in which women must adapt to their new social role (Dziurka et al., 2021) have been studied from different perspectives. Traditionally, perinatal research has focused on emotional disorders, demonstrating an increased risk of depression and anxiety during pregnancy and postpartum (Badr et al., 2021; Battulga et al., 2021). However, recent research has been increasingly exploring the positive aspects of psychological well-being during this period, including life satisfaction. Understanding its evolution across pregnancy and postpartum is crucial, as findings remain inconsistent: some studies report increased well-being due to the expectation and experience of motherhood, while others highlight declines related to hormonal changes, fatigue, financial concerns, and lack of social support (Nelson et al., 2014). High life satisfaction during pregnancy has been associated with lower risks of perinatal depression or anxiety (Quick et al., 2023) suggesting a protective role in maternal mental health. Longitudinal studies indicate a peak in life satisfaction shortly after delivery, followed by a gradual return to baseline within two years postpartum (Dyrdal & Lucas, 2013). Key determinants include sociodemographic variables (e.g., age, economic status), psychological traits, and contextual factors like social support. Pregnant women who perceive greater emotional support report higher life satisfaction (Battulga et al., 2021). In the postpartum period, instrumental support—such as help with childcare and household tasks—also plays a crucial role in enhancing maternal well-being (Mao et al., 2024; Hagaman et al., 2021). A meta-analysis confirms the universal positive effect of social support on subjective well-being during pregnancy (Battulga et al., 2021), whereas anxiety and depressive symptoms weaken life satisfaction (Badr et al., 2021).

These findings highlight the importance of considering both psychological variables and contextual factors when examining life satisfaction trajectories in maternity. Taken together, the literature reveals divergent findings regarding the course of life satisfaction across the perinatal period. This inconsistency underscores the importance of longitudinal approaches that can clarify trajectories within specific sociocultural contexts (Quick et al., 2023). Overall, prior research has primarily examined life satisfaction either in the general population or in the short-term perinatal period, with only a few longitudinal studies extending beyond the first two years postpartum. As a result, there is limited evidence on the long-term trajectories of maternal life satisfaction and the psychological and contextual factors that shape them (Quick et al., 2023; Dyrdal & Lucas, 2013). The Israeli context provides a distinctive sociocultural setting, as familism remains a powerful cultural force while coexisting with individualism and neoliberal demands (Berkovitch & Manor, 2022).

Given the above, the present study aims to analyze the evolution of life satisfaction from pregnancy to five years postpartum, identifying factors that influence this trajectory. Specifically, it examines the role of dispositional characteristics (e.g., education, family income) and clinical-contextual variables (e.g., social support, attachment style, self-efficacy). By addressing existing gaps in the literature, this study aims to contribute empirical insights into psychosocial factors that may be associated with long-term maternal well-being, offering a basis for designing interventions that promote life satisfaction and psychological health in mothers during the transition to motherhood and early child-rearing years.

## 2. Materials and Methods

### 2.1. Design

A prospective longitudinal study was carried out with four data collection periods: the third trimester of pregnancy (T1), eight weeks after birth (T2), five months after birth (T3) and five years after birth (T4). During the fourth time period, the evolution of participants’ life satisfaction was assessed. Sociodemographic and psychosocial variables were evaluated only at the first time point (T1) (Table 1). The final sample (at time point 4) consisted of 231 women. There were *n* = 549 women at time point 1, *n* = 471 at time point 2 and *n* = 352 at time point 3.

### 2.2. Procedure and Participants

The research team developed an ad hoc questionnaire to collect data, incorporating all the variables under study and attaching various validated instruments. Participants were required to provide informed consent. Additionally, those interested in participating in later phases of the study were asked to supply their email addresses.

Women in their third trimester of pregnancy were recruited from Public Health Services in eight cities in central Israel through a convenience consecutive sampling strategy during routine prenatal visits. Recruitment was conducted by the research team, who explained the study and obtained informed consent. Eligibility criteria included being 18 years of age or older and Hebrew proficiency. For the follow-up phases, participants were recontacted by the research team via email, text messages, and phone calls.

A final sample of 231 women participated in the four phases of the research. Initially, a minimum number of *n* = 120 was considered as the sample size for methodological criteria. An a priori power analysis was conducted using G*Power version 3.1.9.7. for a linear regression model with six predictors, assuming a medium effect size (f^2^ = 0.15), α = 0.05, and power = 0.90. The required sample size was estimated to be 98 participants. Additionally, according to Green (1991), a minimum of *n* ≥ 50 + 8 m (number of predictor variables) is recommended for testing linear regression model, further supporting the adequacy of the sample.

In the initial phase of the research (T1), a total of 549 pregnant women participated, who were invited via email and text message to complete the follow-up questionnaires. Of these, 471 participated at time point 2, and 352 at time point 3. The attrition rate was 57.9% from T1 to T4, which is considered reasonable for a longitudinal study of this duration (Friesen et al., 2017). Factors contributing to attrition included difficulties in locating participants, concerns about providing further personal information, and limited time or availability. Comparisons were conducted between participants who remained in the study at T4 and those who dropped out. No significant differences were observed in baseline variables, indicating that attrition did not introduce bias into the results.

Ethical approval was obtained from the Ethics Committee of Tel Aviv University (07122008TAU-SOC-KG, 7 December 2008) and from the Helsinki Committees (IRBs) of the Israeli Health Services: Clalit (39/09k, 6 April 2009), Maccabi (2008073, 19 November 2008), and Leumit (06082008LEU-TK, 6 August 2008).

### 2.3. Variables and Instruments

#### 2.3.1. Satisfaction with Life [Time Points 1, 2, 3 and 4]

The Satisfaction with Life Scale (SWLS) (Diener et al., 1985) was used. It is a five-item measure designed to evaluate overall life satisfaction. Participants indicated their level of agreement with each item using a 7-point Likert scale, ranging from 1 (strongly disagree) to 7 (strongly agree). The total score was calculated by averaging the responses, with higher scores reflecting greater life satisfaction (Diener et al., 1985). The scale demonstrated high reliability (α = 0.85).

#### 2.3.2. Sociodemographic and Occupational Variables [Time Point 1]

An ad hoc questionnaire developed by the research team was used to collect data. Specifically, these data were age, number of years of education, family situation (married, single, separated/divorced), employment type (full time/part-time/not working at the moment), family income relative to the average income in Israel (distinguishing between participants below, about the average or above), number of children (from waiting for first child, or the option of one, two or more than three), whether it was a planned pregnancy (yes/no), and whether they became pregnant in a natural way (yes/no).

#### 2.3.3. Psychosocial Variables [Time Point 1]

- Positive and Negative Affect were assessed using the Positive and Negative Affect Schedule (Watson et al., 1988). The Positive Affect (PA) and Negative Affect (NA) scales each consist of 10 items. Participants are required to indicate the extent to which they have experienced each affect using a five-point Likert scale (from 1 = not at all or very slightly to 5 = very much) during the specified time period (in this case, in the past month). The total score for each scale is the sum of the ratings given to the 10 items, ranging from 10 to 50 points, with higher scores indicating a greater presence of the specific affect. The Cronbach’s alpha was 0.87 for positive affect and 0.86 for negative affect. Positive and negative affect are central components of emotional functioning and subjective well-being. As highlighted in recent meta-analytic study, these affective dimensions are closely linked to life satisfaction and provide a nuanced understanding of emotional experiences across the lifespan, including during motherhood (Buecker et al., 2023).
- Social support. Perceived social support was measured using a 9-item scale (House, 1981; Westman et al., 2004) to assess three sources of support: spouse, family, and friends. Participants indicated the extent to which they: (a) shared their recent experiences with each source, (b) received emotional support and understanding, and (c) obtained advice or instrumental assistance. Responses were recorded on a 5-point Likert scale, ranging from 1 (not at all) to 5 (very much). Higher average scores indicated greater perceived social support. The scale demonstrated good internal consistency (Cronbach’s alpha = 0.81).
  The decision to use this scale was guided by both theoretical and methodological considerations. While more comprehensive instruments such as the Medical Outcomes Study Social Support Survey (MOS-SSS) are available, the brevity of the 9-item scale was deemed more appropriate given the multi-instrument nature of the survey and the need to reduce participant fatigue. The scale was used in its Hebrew version (Westman et al., 2004), without adaptation, and its validity has been supported in previous research (Lavenda & Kestler-Peleg, 2017; Kestler-Peleg & Lavenda, 2021). Social support is a widely recognized protective factor in maternal mental health. Recent qualitative reviews emphasize its role in buffering stress and enhancing resilience during pregnancy and early motherhood (Al-Mutawtah et al., 2023).
- Attachment (anxiety and avoidance). Adult attachment style was measured using the Experience in Close Relationships Inventory (Brennan et al., 1998). This 36-item scale evaluates personal feelings toward close relationships on a 7-point Likert scale, ranging from 1 (strongly disagree) to 7 (strongly agree). It assesses two key dimensions: avoidance (e.g., “I try to avoid getting too close to my partner”) and anxiety (e.g., “I worry a lot about my relationships”). Higher scores indicate a stronger tendency toward avoidant or anxious attachment. The scale demonstrated high reliability, with a Cronbach’s alpha of 0.88 for anxiety and 0.89 for avoidance. Attachment, particularly the emotional bond between mother and child, plays a key role in shaping maternal identity and psychological stability. Recent findings support its predictive value for life satisfaction and emotional well-being (Martucci et al., 2021).
- Maternal self-efficacy was measured using the Parenting Sense of Competence Questionnaire (PSOC) (Johnson & Mash, 1989). This 18-item scale evaluates a mother’s perception of her effectiveness in fulfilling her parental role (e.g., “I have all the skills necessary to be a good mother to my child”). Participants rated their level of agreement with the statements concerning their children using a 4-point Likert scale ranging from 1 (not at all) to 4 (very much). Higher average scores indicate greater maternal self-efficacy. The scale demonstrated good internal consistency, with a Cronbach’s alpha of 0.85. Maternal self-efficacy has been shown to influence emotional outcomes and parenting satisfaction. Longitudinal research confirms its development from pregnancy through the postpartum period and its relevance for maternal adjustment (Samdan et al., 2022).

### 2.4. Data Analysis

Descriptive analyses and Cronbach’s alpha were conducted. Categorical variables were summarized using frequencies (*n*) and percentages (%), while continuous variables were reported as means (M) and standard deviations (SD). To examine the bivariate relationships between variables and identify potential covariates, different statistical tests were applied based on the nature of the data: Student’s t-test, one-way analysis of variance (ANOVA), and Pearson’s correlation. A linear regression analysis was performed to assess the contribution of psychosocial variables (evaluated at T1) in Satisfaction with Life (SWL) at the different time points considered (T1–T4), employing the stepwise method for predictor variable selection. Subgroup analyses were also conducted based on employment status and parity. Statistical analyses were carried out using the Statistical Package for the Social Sciences (SPSS), version 21 for Windows. A significance level of *p* < 0.05 was considered for all analyses. In line with the exploratory nature of this study, we employed longitudinal analyses across four time points without applying theory-driven hierarchical modeling or formal model selection criteria such as AIC or BIC. This decision was based on the limited availability of prior longitudinal research addressing the specific variables and time frames examined. Our primary aim was to identify general temporal patterns and associations, rather than to test a predefined theoretical model. This approach allowed for flexibility in detecting emerging trends and relationships that may inform future hypothesis-driven research.

## 3. Results

### 3.1. Description of the Sociodemographic and Occupational Variables of the Sample

Table 2 shows the sociodemographic and occupational data of the 231 women, represented by frequencies, percentages, means, and SD. All data refer to time T1 (third trimester of pregnancy). The final sample consisted of 231 Israeli mothers, aged between 27 and 51 years (M = 37.10, SD = 4.34), with an educational background ranging from 11 to 22 years (M = 16.46, SD = 4.34). The majority of the participants were married or cohabiting with a partner (96.1%), while a small percentage were single (0.9%), divorced or separated (3%). Among the mothers, about 40% were waiting for their first child. Almost all of the women had planned their pregnancies and had natural pregnancy. Regarding employment status, more than 80% were working in their third trimester of pregnancy, the vast majority of them full-time. In terms of income, most participants (about 70%) perceived their economic situation as above average in Israel.

### 3.2. Associations Between Satisfaction with Life (SWL) and Sociodemographic, Occupational and Psychosocial Variables of the Sample

Table 2 summarizes the associations between various sociodemographic characteristics evaluated at T1 and satisfaction with life measured at four time points (T1–T4). Age was found to be significantly and negatively related to SWL at T2 and T4, indicating that older mothers tended to report slightly lower life satisfaction. Education showed a positive association with SWL only at T1. Moreover, employment status at T1 is related to SWL at T1 and T2, with mothers working full-time or part-time reporting higher scores compared to those who were not employed. A higher income was consistently linked to higher SWL at T1, T2, and T3. The number of children, as well as whether the pregnancy was planned or natural, did not show statistically significant relationships with SWL across the different time points.

Table 3 displays the relationships between various psychosocial variables measured at time 1 (T1)—including positive affect, negative affect, social support, avoidance in engagement, attachment anxiety, and maternal self-efficacy—and satisfaction with life assessed at four different time points (T1–T4). Overall, positive affect, social support, and maternal self-efficacy at T1 showed significant positive associations with satisfaction with life at all four time points. In contrast, negative affect, avoidance in engagement, and attachment anxiety at T1 were negatively related to satisfaction with life across T1–T4. The strength of these relationships (as indicated by *r*^2^) remained consistently significant. Specifically, positive affect and social support demonstrated moderate to strong positive correlations with satisfaction with life, whereas negative affect, avoidance in engagement, and attachment anxiety displayed moderate negative correlations. Notably, maternal self-efficacy showed higher correlation coefficients at later time points (T3 and T4), indicating that confidence in one’s maternal role at the end of pregnancy may become increasingly important for life satisfaction as time progresses. When interpreting these results, it is important to keep in mind, as noted, that both the sociodemographic and psychosocial variables were only assessed at T1, so the associations of these variables with life satisfaction measured at other time points (T2–T4) are based on baseline data. Although, given the nature of the variables (associated with personal traits), a certain temporal stability can be assumed, this stability is questionable over the duration of the study (5 years).

### 3.3. Evolution of Satisfaction with Life

**Figure 1:**
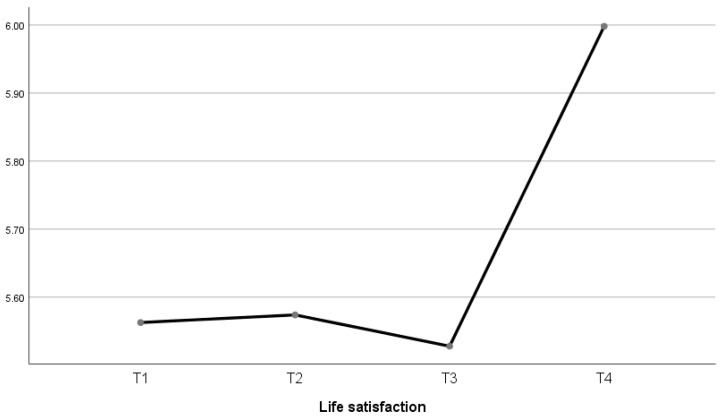
Satisfaction with life averages for the sample at the different time points.

The sample presented the highest mean score for the satisfaction with life scale (5.99; SD = 0.12) at the fourth time point. It can be observed (Table 4 and Figure 1) that, while at first and second time points, satisfaction with life is maintained, it takes a downward turn at the third time point (5.52; SD = 0.08) and then changes five years after birth (time point 4). Statistically significant differences were observed between time points 1 and 4 and between time points 2 and 4, as well as between time points 3 and 4. 

### 3.4. Linear Regression Analysis Considering Life Satisfaction as the Outcome and Psychosocial Variables as Predictors

A linear regression using a stepwise approach was conducted to examine the influence of psychosocial variables—measured only at baseline (T1)—on life satisfaction assessed at each of the four time points. Psychosocial variables considered include positive affect, negative affect, social support, avoidant and anxious attachment, and maternal self-efficacy. The final models are presented in Table 5. The model explained 17% of the variance at T1, 11% of the variance at T2, 14% of the variance at T3 and 17% of the variance at T4. At T1, positive affect (*p* = 0.030), negative affect (*p* = 0.007), avoidance in engagement (*p* < 0.001), attachment anxiety (*p* = 0.008), and maternal self-efficacy (*p* = 0.041) emerged as significant predictors, while social support did not show a significant effect. At T2, social support was the only significant predictor (*p* = 0.019). At T3, positive affect (*p* = 0.033) and attachment anxiety (*p* = 0.001) were significant predictors. Finally, at T4, social support (*p* = 0.007) and avoidance in engagement (*p* = 0.005) were significantly associated with satisfaction with life.

### 3.5. Subgroup Analysis: Employment Status and Parity

Given the sample distribution in terms of sociodemographic variables (see Table 2), an additional regression analysis was proposed, considering the existence of possible differences in the subgroups. Of the sociodemographic variables evaluated (all of them at T1), complementary regression analyses were performed by subgroups with two of them: Employment Status (Working vs. Not Working) and Parity (First-time vs. Multiparous Mothers). The selection of these variables was based on methodological (greater heterogeneity in our sample) and conceptual reasons. Regarding the latter, employment status during pregnancy is a critical factor influencing maternal well-being. Working mothers may benefit from social engagement and financial stability, but also face challenges related to work–life balance and role overload. Conversely, non-working mothers may experience isolation or financial stress, both of which can affect psychological outcomes (Aochi et al., 2021; Kim & Wickrama, 2021). On the other hand, parity has been consistently associated with differences in maternal psychological adjustment, coping strategies, and perceived support. First-time mothers often face greater uncertainty, higher anxiety levels, and more pronounced shifts in identity, which can influence their satisfaction with life and the impact of psychosocial variables such as self-efficacy, affect, and attachment. In contrast, multiparous women may benefit from prior experience, but also face cumulative stressors, especially when managing multiple children (Prosen & Ličen, 2025; Vaillancourt et al., 2022).

Table 6 presents the significance levels of psychosocial variables (T1) as predictors of life satisfaction (T1–T4) within subgroups defined by employment status, while Table 7 shows the same analysis conducted within subgroups based on parity. The differences (statistically significant and non-significant) with respect to the regression analyses performed with the entire sample (see previous Section 3.4) appear in italics.

As can be seen, subgroup analyses conducted with the variables employment status (working vs. not working) and parity (first-time mothers vs. multiparous mothers) show differential effects with respect to the general trend (see previous Section 3.4). Specifically for employment status, with regard to life satisfaction at T1, differential effects are observed for positive affect and self-efficacy (non-working women) and for negative affect and attachment (working women). At T2, differences are observed in the role of social support and avoidant attachment (non-working women) and in positive affect and self-efficacy (working women). At T3, differential effects are only observed in non-working women for positive affect and anxious attachment. Finally, at T4, differential effects are observed for social support (non-working women) and for social support and avoidant attachment (working women). Subgroup analyses performed on the variable “Parity” also showed differential results. For the prediction of life satisfaction at T1, differences with respect to the general pattern are observed in all variables except social support (first-time mothers) and attachment (multiparous mothers). At T2, differences are observed in social support (first-time mothers) and in social support and positive affect (multiparous mothers). At T3, differences are observed in anxious attachment and positive affect (first-time mothers) and in positive affect (multiparous mothers). Finally, at T4, differences are observed in social support and avoidant attachment (first-time mothers) and in avoidant attachment (multiparous mothers).

## 4. Discussion

This research examined the evolution of life satisfaction during different stages of motherhood—from the third trimester of pregnancy to five years postpartum—considering key psychological factors evaluated at baseline and the sociocultural context of Israel. In the third trimester of pregnancy, life satisfaction was found to be significantly negatively correlated with negative affect and insecure attachment styles, both avoidant and anxious. These results are consistent with the literature linking insecure attachment styles with lower well-being and greater emotional distress (Mikulincer & Shaver, 2010). Given that pregnancy is a time of high emotional sensitivity (Ankit & Jyotsna, 2022), it is not surprising that women with insecure attachment patterns experience lower life satisfaction, especially in a society like Israel, where family and community ties are fundamental (Katz et al., 2010). In this context, the inability to fully benefit from cultural social support systems can exacerbate the emotional difficulties of expectant mothers (Katz et al., 2010). At eight weeks postpartum, life satisfaction levels remained similar to those in the third trimester, but the dynamics of the relationships changed. At this stage, a positive and significant association was found between life satisfaction and social support, reinforcing the protective role of support networks in maternal well-being (Kay et al., 2024). Beyond its immediate effect, this association is consistent with broader evidence showing that support contributes to maternal adjustment not only directly but also indirectly through relational pathways. For instance, perceived support has been shown to alleviate postpartum stress by enhancing marital satisfaction and strengthening the mother–infant bond (Wang et al., 2023). Such relational mechanisms may help explain the robust associations between social support and maternal life satisfaction documented across studies. Furthermore, a trend toward a negative correlation was observed between life satisfaction and avoidant attachment, as well as a positive association with maternal self-efficacy. In Israel, where government policies support mothers through paid maternity leave and childcare benefits (Rubin et al., 2017), the availability of social support is especially crucial in the early stages of motherhood. However, women with avoidant attachment may not fully seek or benefit from this support, which could explain lower levels of life satisfaction in some cases (Mikulincer & Shaver, 2010).

At five months postpartum, life satisfaction was found to be positively correlated with positive affect and negatively correlated with anxious attachment. This finding suggests that, as mothers progress through the postpartum period, their ability to regulate their emotions and maintain a positive affective state becomes a determining factor in their well-being (Field, 2010). Israeli culture, with its strong emphasis on family values and extended intergenerational support networks (Lavee & Katz, 2003), facilitates the integration of mothers into support systems that can enhance their well-being, provided they are able to experience positive emotions. However, mothers with an anxious attachment style tend to feel overwhelmed by intense emotions, which can impair their ability to adapt to the demands of the postpartum period, especially when insecurities about their mothering performance arise, as is common among first-time mothers or those with multiple children (Berkovitch, 1997).

Finally, at five years postpartum, life satisfaction again showed a strong positive association with social support and a negative relationship with avoidant attachment. Notably, women with avoidant attachment styles reported significantly lower levels of life satisfaction, while those with high levels of social support experienced greater satisfaction. Prior studies suggest that the protective role of support is shaped not only by its availability but also by the quality and continuity of the relational context in which it is provided. Well-functioning family dynamics have been found to amplify the benefits of support (Huang et al., 2021), while continuous emotional support across the perinatal period plays a particularly critical role in reducing maternal distress and promoting adaptation (Hagaman et al., 2021). Taken together, these relational and emotional dimensions highlight the long-term impact of both interpersonal and environmental factors on maternal life satisfaction. In a context where Israeli policies and cultural norms encourage communal parenting (Birenbaum-Carmeli, 2009; Lavee & Katz, 2003) and provide institutional support through early education programs and social benefits (Rubin et al., 2017), the sustained presence of support networks appears to act as a critical buffer against declining life satisfaction over time. However, the reluctance of some avoidant mothers to draw on these resources may contribute to persistently low life satisfaction, negatively affecting their motherhood experience even years after childbirth (Field, 2010).

Taken together, these findings reinforce the complexity and multifaceted nature of life satisfaction during motherhood, highlighting the interaction between psychological predispositions—such as attachment styles and affective state—and contextual factors, primarily social support. The results align with previous studies demonstrating that social support has a consistent and positive effect on maternal satisfaction, whereas insecure attachment patterns, particularly avoidant attachment, pose a risk to long-term satisfaction (Berkovitch, 1997; Mikulincer & Shaver, 2010). Furthermore, these findings take on particular relevance in the context of Israel, a society with strong pro-natalist policies and a cultural emphasis on the family (Berkovitch, 1997; Birenbaum-Carmeli, 2009; Lavee & Katz, 2003). Overall, our findings underscore that maternal life satisfaction is shaped by a complex interplay of psychological dispositions, relational processes, and sociocultural contexts, with implications for both research and interventions that extend well beyond the immediate postpartum period.

From a clinical perspective, these findings underscore the importance of integrating psychosocial screening and support into perinatal care. Identifying attachment-related vulnerabilities during pregnancy may help clinicians recognize women at higher risk of reduced life satisfaction. In addition, routine assessment of maternal self-efficacy and perceived social support could guide tailored interventions. Structured peer-support programs, parental education, and counseling focused on enhancing self-efficacy and promoting the effective use of social support networks may serve as protective resources across the transition to motherhood. Embedding such approaches into perinatal services has the potential to improve maternal adjustment and promote sustained life satisfaction in the years following childbirth.

This study presents several limitations that should be acknowledged. Conceptual limitations include the timing and scope of data collection. Although the perinatal period begins in the second trimester and ends shortly after birth, data collection started in the third trimester and did not include assessments immediately postpartum, limiting the ability to capture early and acute psychological changes. Additionally, all psychosocial predictor variables (e.g., affect, self-efficacy, attachment) were assessed only once during pregnancy. While these constructs are generally considered stable in the short term, the five-year span of the study introduces the possibility of change over time, which could have influenced life satisfaction differently across the postpartum period. Future research should incorporate repeated assessments to better understand the dynamic role of these variables in maternal well-being.

Design-related limitations include the use of static predictors in a longitudinal framework, evaluated only at T1, which restricts the modeling of dynamic trajectories of maternal life satisfaction. Moreover, the study did not track changes in sociodemographic or psychological characteristics over time, limiting the validity of causal inferences. Unmeasured variables—such as coping strategies or cultural influences—may also have played a role, underscoring the need for broader and more flexible designs in future research.

Methodological limitations concern the analytical approach. The study employed exploratory stepwise regression without formal model selection criteria (e.g., AIC or BIC), which may affect the precision and generalizability of the findings. Subgroup analyses were limited to parity and employment status to maintain model parsimony, although future studies could benefit from exploring additional contextual moderators.

Some limitations related to the sample characteristics should also be considered when interpreting the findings. The sample was not fully representative of the general population, as most participants were married, highly educated, and from higher-income backgrounds. This homogeneity limits the generalizability of the findings, particularly to underrepresented groups such as single mothers, same-sex couples, immigrants, and families from lower socioeconomic backgrounds. Given that subgroup analyses revealed differential influences of psychosocial variables based on economic status and parity, future studies should aim to recruit more diverse populations to examine whether life satisfaction trajectories vary across sociodemographic and cultural contexts.

Finally, participant attrition over time—57.9% from the first to the fourth time point—may have influenced the results and reduced statistical power in later phases. Although comparative analyses of baseline variables (e.g., positive and negative affect, attachment, social support and maternal self-efficacy) showed no significant differences between completers and dropouts, suggesting minimal bias, future studies should implement strategies to reduce attrition and ensure consistent participation.

## 5. Conclusions

Despite the above limitations, the present study highlights variations in maternal life satisfaction observed between pregnancy and postpartum, underscoring the role of psychological and contextual factors. Maternal life satisfaction across the study period was associated with baseline attachment style, affect, perceived social support, and maternal self-efficacy. Insecure attachment styles (avoidant and anxious) were consistently linked to lower satisfaction, whereas robust social support acted as a protective factor. In the Israeli context—where motherhood is highly valued and supported by strong familial and institutional structures—these findings may hold particular relevance. Women with avoidant attachment may not fully benefit from available support, underscoring the need for targeted interventions.

Practically, these findings emphasize the importance of integrating psychosocial considerations into perinatal care, with the potential to guide preventive interventions and inform public health policies. Overall, this study integrates psychological and environmental perspectives to improve our understanding of maternal life satisfaction. Despite differences in specific groups (i.e., based on employment status and parity), the results highlight the importance of assessing and addressing these psychosocial variables, both protective and risk factors. Future research is encouraged to incorporate repeated assessments of predictor variables, conduct stratified analyses to account for sociodemographic differences, examine causal pathways linking attachment, support, and life satisfaction, and explore culturally specific factors that may shape maternal life satisfaction. Such approaches could better inform personalized care strategies and public health policies.

## Figures and Tables

**Table 1:** Variables collected at different time points.

Variables Collected at Different Time Points				
	1st Evaluation Period	2nd Evaluation Period	3rd Evaluation Period	4th Evaluation Period
	Third Trimester	Eight Weeks After Birth	Five Months After Birth	Five Years After Birth
Outcome Variable	Satisfaction with Life	Satisfaction with Life	Satisfaction with Life	Satisfaction with Life
Sociodemographic	Age, years of education, marital status, working status, scope of employment, family income, number of children, planned pregnancy and natural pregnancy.			
Psychosocial variables	Positive and Negative Affect Social support Attachment (anxiety and avoidance) Maternal self-efficacy			

**Table 2:** Sociodemographic characteristics and their associations with Satisfaction with Life (SWL).

				Satisfaction with Life (SWL)												
				Time Moment 1				Time Moment 2			Time Moment 3			Time Moment 4		
		f (%)	Mean (SD)	Mean (SD)	Test		*p*	Mean (SD)	Test	*p*	Mean (SD)	Test	*p*	Mean (SD)	Test	*p*
Age			37.10 (4.34)		$r^{2}$	−0.035	0.421		−0.147	0.002		−0.046	0.412		−0.176	0.007
Education			16.46 (1.97)		$r^{2}$	0.093	0.035		0.023	0.628		0.021	0.704		0.107	0.104
Working	Full time	347 61.5%		5.45 (0.89)	F	6.087	0.002	5.55 (0.87)	4.133	0.017	5.52 (0.95)	0.475	0.623	5.87 (1.34)	2.242	0.109
	Part-time	125 22.2%		5.60 (0.89)				5.69 (0.74)			5.50 (0.96)			6.25 (1.61)		
	Not working	92 16.3%		5.14 (1.20)				5.29 (1.04)			5.37 (1.08)			5.63 (1.64)		
Income	Below average	70 12.5%		5.17 (1.19)	F	7.385	0.001	5.45 (0.90)	3.832	0.022	5.21 (0.96)	5.214	0.006	6.00 (1.32)	1.510	0.223
	About average	124 22.2%		5.28 (1.07)				5.36 (1.08)			5.23 (1.16)			5.58 (1.68)		
	Above average	364 65.2%		5.56 (0.82)				5.63 (0.79)			5.61 (0.90)			5.98 (1.43)		
Number of children	Waiting for first child	204 38.9%		5.46 (0.96)	F	1.443	0.230	5.58 (0.95)	1.970	0.118	5.44 (1.03)	0.870	0.45	5.96 (1.38)	0.594	0.620
	One	176 33.5%		5.33 (1.02)				5.38 (0.99)			5.41 (1.03)			5.76 (1.51)		
	Two	106 20.2%		5.48 (0.84)				5.63 (0.75)			5.60 (0.89)			5.85 (1.68)		
	Three or more	39 7.4%		5.67 (0.85)				5.66 (0.82)			5.48 (0.99)			6.27 (1.23)		
Planned pregnancy	Yes	543 91.3%		5.44 (0.95)	T	1.449	0.148	5.55 (0.89)	1.732	0.084	5.51 (0.95)	0.767	0.444	5.89 (1.45)	1.041	0.299
	no	52 8.7%		5.23 (1.04)				5.28 (1.06)			5.36 (1.22)			5.48 (2.10)		
Natural Pregnancy	yes	543 90.7%		5.43 (0.97)	T	0.438	0.661	5.53 (0.90)	0.774	0.439	5.50 (0.99)	0.816	0.415	5.89 (1.49)	0.865	0.388
	No	56 9.3%		5.37 (0.94)				5.43 (0.90)			5.35 (0.87)			5.62 (1.49)		

**Table 3:** Psychosocial variables and their associations with Satisfaction with Life (SWL).

			Satisfaction with Life (SWL)												
			Time Point 1				Time Point 2			Time Point 3			Time Point 4		
	f (%)	Mean (SD)	Mean (SD)	Test		*p*	Mean (SD)	Test	*p*	Mean (SD)	Test	*p*	Mean (SD)	Test	*p*
Positive Affect T1		3.33 (0.65)		$r^{2}$	0.237	<0.001		0.224	<0.001		0.232	<0.001		0.229	<0.001
Negative Affect T1		2.18 (0.64)		$r^{2}$	−0.276	<0.001		−0.204	<0.001		−0.192	<0.001		−0.179	0.007
Social support T1		3.90 (0.55)		$r^{2}$	0.232	<0.001		0.241	<0.001		0.211	<0.001		0.366	<0.001
Avoidance in engagement T1		2.85 (0.77)		$r^{2}$	−0.261	<0.001		−0.182	<0.001		−0.168	0.002		−0.333	<0.001
Attachment anxiety T1		2.88 (0.81)		$r^{2}$	−0.272	<0.001		−0.221	<0.001		−0.298	<0.001		−0.260	<0.001
Maternal self-efficacy T1		3.22 (0.36)		$r^{2}$	0.195	<0.001		0.23	<0.001		0.251	<0.001		0.309	<0.001

**Table 4:** Satisfaction with Life (SWL) at each of the time points of data collection.

					Student’s Test for Paired Samples											
	Time 1	Time 2	Time 3	Time 4	Time 1–2		Time 1–3		Time 1–4		Time 2–3		Time 2–4		Time 3–4	
	M (SD)	M (SD)	M (SD)	M (SD)	t	*p*	t	*p*	t	*p*	t	*p*	t	*p*	t	*p*
Satisfaction with Life	5.56 (0.07)	5.57 (0.07)	5.52 (0.08)	5.99 (0.12)	−0.766	0.444	1.387	0.166	−5.924	<0.001	1.729	0.085	−4.873	<0.001	−4.525	<0.001

**Table 5:** Linear regression analysis considering life satisfaction as the outcome (including different psychological variables).

Satisfaction with Life	F	$R^{2}$	*p*	Beta	t	*p*
Satisfaction with Life T1	17.98	0.170	<0.001			
Positive Affect				0.100	2.181	0.030
Negative Affect				−0.127	−2.726	0.007
Social support				0.052	1.158	0.247
Avoidance in engagement				−0.176	−4.124	<0.001
Attachment anxiety				−0.120	−2.666	0.008
Maternal self-efficacy				0.091	2.047	0.041
Satisfaction with Life T2	9.53	0.116	<0.001			
Positive Affect				0.088	1.687	0.092
Negative Affect				−0.069	−1.310	0.191
Social support				0.119	2.352	0.019
Avoidance in engagement				−0.091	−1.878	0.061
Attachment anxiety				−0.086	−1.661	0.097
Maternal self-efficacy				0.092	1.819	0.070
Satisfaction with Life T3	8.908	0.143	<0.001			
Positive Affect				0.126	2.139	0.033
Negative Affect				−0.045	−0.768	0.443
Social support				0.056	0.950	0.343
Avoidance in engagement				−0.075	−1.306	0.193
Attachment anxiety				−0.200	−3.428	0.001
Maternal self-efficacy				0.091	1.591	0.113
Satisfaction with Life T4	7.418	0.168	<0.001			
Positive Affect				0.054	0.742	0.459
Negative Affect				−0.008	−0.102	0.919
Social support				0.190	2.747	0.007
Avoidance in engagement				−0.193	−2.850	0.005
Attachment anxiety				−0.120	−1.604	0.110
Maternal self-efficacy				0.024	0.329	0.742

**Table 6:** Subgroup analysis: Employment status (working vs. not working).

	T1		T2		T3		T4	
	No	Yes	No	Yes	No	Yes	No	Yes
Positive Affect	*0.597*	0.003	0.444	*0.035*	*0.525*	0.049	0.598	0.127
Negative Affect	0.002	*0.397*	0.334	0.331	0.437	0.243	0.408	0.607
Social support	0.480	0.128	*0.518*	0.042	0.320	0.084	*0.663*	*0.070*
Avoidance in engagement	0.005	*0.165*	*0.038*	0.816	0.968	0.731	0.008	*0.190*
Attachment anxiety	0.041	*0.098*	0.383	0.211	*0.092*	0.025	0.881	0.120
Maternal self-efficacy	*0.160*	0.025	0.229	*0.040*	0.124	0.154	0.154	0.801

No (not working), yes (working). Italicized values denote a shift in statistical significance status relative to the full-sample regression model (see Section 3.4); specifically, predictors that were significant in the full sample but non-significant in the subsample, and vice versa.

**Table 7:** Subgroup analysis: Parity (first-time mothers vs. multiparous mothers).

	T1		T2		T3		T4	
	1	2	1	2	1	2	1	2
Positive Affect	*0.202*	0.019	0.650	*0.046*	*0.272*	*0.169*	0.778	0.053
Negative Affect	*0.106*	0.040	0.283	0.154	0.217	0.333	0.538	0.714
Social support	0.556	0.544	*0.128*	*0.341*	0.095	0.485	*0.196*	0.049
Avoidance in engagement	*0.152*	*0.064*	0.378	0.251	0.972	0.470	*0.172*	*0.233*
Attachment anxiety	*0.148*	*0.098*	0.826	0.066	*0.495*	0.004	0.365	0.295
Maternal self-efficacy	*0.317*	0.024	0.085	0.363	0.236	0.499	0.995	0.854

1 (first-time mothers), 2 (multiparous mothers). Italicized values denote a shift in statistical significance status relative to the full-sample regression model (see Section 3.4); specifically, predictors that were significant in the full sample but non-significant in the subsample, and vice versa.

## Data Availability

The data presented in this study are available on request from the corresponding author. The data are not publicly available due to privacy restrictions.
